# Sensitivity and specificity of metatranscriptomics as an arbovirus surveillance tool

**DOI:** 10.1038/s41598-019-55741-3

**Published:** 2019-12-18

**Authors:** Jana Batovska, Peter T. Mee, Stacey E. Lynch, Tim I. Sawbridge, Brendan C. Rodoni

**Affiliations:** 1 Agriculture Victoria Research, AgriBio Centre for AgriBioscience, Bundoora Victoria, Australia; 2 School of Applied Systems Biology, La Trobe University, Bundoora Victoria, Australia

**Keywords:** RNA sequencing, Metagenomics, Pathogens, Alphaviruses, Classification and taxonomy

## Abstract

The ability to identify all the viruses within a sample makes metatranscriptomic sequencing an attractive tool to screen mosquitoes for arboviruses. Practical application of this technique, however, requires a clear understanding of its analytical sensitivity and specificity. To assess this, five dilutions (1:1, 1:20, 1:400, 1:8,000 and 1:160,000) of Ross River virus (RRV) and Umatilla virus (UMAV) isolates were spiked into subsamples of a pool of 100 *Culex australicus* mosquitoes. The 1:1 dilution represented the viral load of one RRV-infected mosquito in a pool of 100 mosquitoes. The subsamples underwent nucleic acid extraction, mosquito-specific ribosomal RNA depletion, and Illumina HiSeq sequencing. The viral load of the subsamples was also measured using reverse transcription droplet digital PCR (RT-ddPCR) and quantitative PCR (RT-qPCR). Metatranscriptomic sequencing detected both RRV and UMAV in the 1:1, 1:20 and 1:400 subsamples. A high specificity was achieved, with 100% of RRV and 99.6% of UMAV assembled contigs correctly identified. Metatranscriptomic sequencing was not as sensitive as RT-qPCR or RT-ddPCR; however, it recovered whole genome information and detected 19 other viruses, including four first detections for Australia. These findings will assist arbovirus surveillance programs in utilising metatranscriptomics in routine surveillance activities to enhance arbovirus detection.

## Introduction

Metatranscriptomics (total RNA sequencing) enables nontargeted, high-throughput detection and characterisation of viruses in a sample. It can be used to detect both known and novel viruses while providing whole genome information, making it a powerful surveillance tool. Metatranscriptomics has been used in a range of surveillance situations, including detecting viruses in human sewage^1^, monitoring viruses in invertebrate vectors such as ticks^2^ and vertebrate reservoirs such as bats^3^, and tracking virus strains during an outbreak^4^. The successful utilisation of metatranscriptomics in a range of surveillance applications suggests it has potential to enhance current arbovirus (arthropod-borne virus) surveillance programs.

Arboviruses represent a significant burden to human and animal health and include pathogens such as dengue, yellow fever, Zika, chikungunya, bluetongue and equine encephalitis viruses, with dengue virus alone infecting an estimated 390 million people per year^5^. Surveillance programs act as an early warning system for increased transmission risk and enlist tools such as mosquito trapping, virus isolation in cell culture, and targeted molecular virus detection using quantitative PCR (qPCR) assays^6–8^. Metatranscriptomics is a nontargeted method that offers many advantages for arbovirus surveillance programs. It can detect viruses without culturing them, does not require *a priori* knowledge of the viral sequence, has the potential to identify new arboviral threats, elucidates mixed infections, and can provide whole genome or specific protein sequences for molecular epidemiological investigations of outbreaks^9^. Furthermore, it can detect other organisms in a mosquito pool, including endosymbionts such as *Wolbachia*^10^, and parasites such as *Leishmania*^11^. The capacity to screen large pools of mosquitoes simultaneously makes metatranscriptomics scalable to adapt to heightened vector abundance^12^.

In order to use metatranscriptomics for arbovirus surveillance, the sensitivity and specificity of the method when testing pools of mosquitoes must first be established. A number of studies have used a metatranscriptomic approach to detect viruses in individual mosquitoes using Illumina^10,13^, Ion Torrent^14^ and Oxford Nanopore^15^ sequencing. More often, pools of mosquitoes are sequenced, ranging from five specimens^11^ to 6,700 specimens^12^. These studies largely focus on exploring the viral diversity present in various mosquito populations. However, there is a lack of studies looking at gold standard test metrics, such as sensitivity and specificity, of metatranscriptomics when testing large pools of mosquitoes for arbovirus surveillance purposes. This is critical when assessing transmission risk and understanding temporal changes in virus abundance. The relationship between viral load and sequencing output needs to be well-defined in order to avoid inaccurate interpretations of sequence data that lead to false positive results (detecting a virus that is not present in the mosquito pool) and false negative results (failing to detect a virus that is present in the mosquito pool).

Laboratory workflows can substantially affect the ability of metatranscriptomic sequencing to detect arboviruses in a mosquito pool. A popular way to increase sensitivity is by enriching for arbovirus using size filtration^16^, PEG precipitation^17^ or sequence-independent amplification^12^. While this does increase the number of viral sequences, enrichment can also introduce bias^18,19^. An alternate way to increase the number of viral sequences is by depleting the mosquito RNA, generally by targeting highly abundant ribosomal RNA (rRNA). A variety of rRNA depletion kits are available, however, these are not specific to mosquitoes and so custom probes based on mosquito rRNA sequences need to be generated^20,21^.

The bioinformatic analyses chosen to process the metatranscriptomic reads can also affect sensitivity and specificity. A common method used to detect viruses in a sample is by mapping reads back to viral reference sequences. However, when dealing with short reads this can lead to false positive results if a virus is present with partial sequence homology to a virus of interest^22^. One way to overcome this problem is by performing *de novo* assembly, where short reads are assembled into longer contiguous sequences (contigs), and then comparing these contigs to a database containing viral reference sequences. This approach can improve specificity because longer fragments are taxonomically classified with greater accuracy^23^. Any viruses detected by the contig-based analysis can then be cross-validated by mapping the sample reads back to the virus reference, which will indicate the breadth and depth of coverage of the virus genome by the reads.

A range of other variables can affect the sensitivity and specificity of metatranscriptomic sequencing including the size and structure (monopartite vs. multipartite) of the virus genome, depth of sequencing, accuracy and completeness of the viral reference database, and the level of host background nucleic acid in the sample^22^. Due to these complications, it can be challenging to establish criteria for positive detection of an arbovirus in a mosquito pool compared to methods like PCR, which is a more targeted detection tool and not impacted by these variables in the same way. As with other detection methods, the use of controls in metatranscriptomics can be used to account for these variables and establish criteria for positive detection. For instance, the addition of a negative control sample that does not contain any viruses can be used to detect viral sequences resulting from physical or cross contamination during the laboratory workflow. Sequence data from the negative control sample can then be used to calculate normalised ratios, for instance the reads per million ratio (RPM-r) where the virus RPM of the sample (RPM_sample_) is divided by the virus RPM of the negative control (RPM_neg_). An RPM-r threshold value of 10 has been used to distinguish a true positive detection from contamination for bacteria, fungi and parasites^24^.

**Figure 1:**
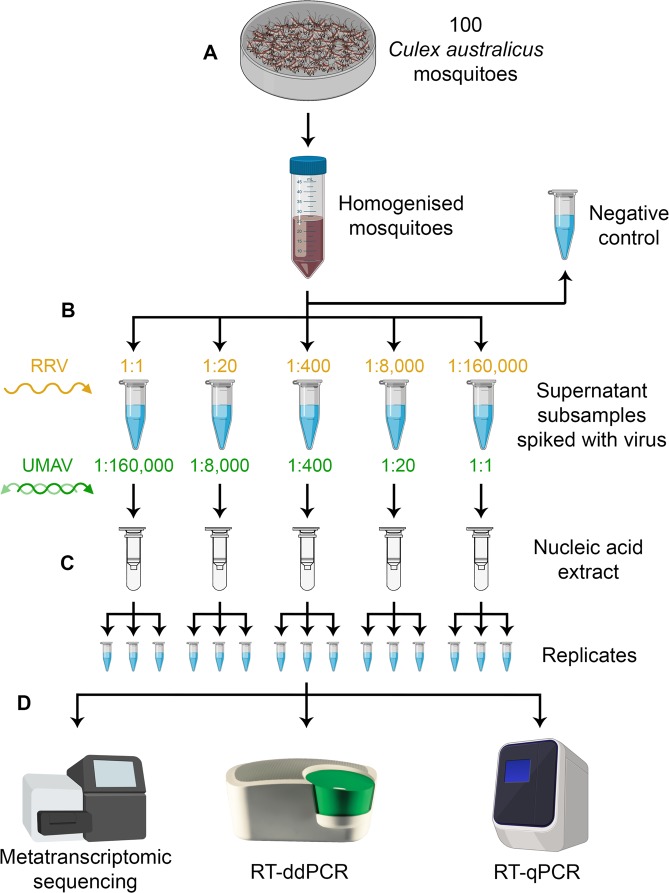
Design of the spiking study. (**A**) 100 mosquitoes were homogenised, centrifuged and the supernatant was subsampled five times, with a sixth subsample taken as a negative control. (**B**) The subsamples were spiked with differing dilutions of Ross River virus (RRV), a monopartite virus, and Umatilla virus (UMAV), a segmented virus. The 1:1 dilution represented the viral load of a single RRV-infected mosquito in a pool of 100. (**C**) Nucleic acid was extracted and split into three technical replicates. (**D**) Viral load was measured using metatranscriptomic sequencing, and reverse transcription droplet digital PCR (RT-ddPCR) and quantitative PCR (RT-qPCR). Created with BioRender.com.

The purpose of this study is to investigate the analytical sensitivity and specificity of a metatranscriptomic pipeline to detect RNA viruses in mosquito pools for arbovirus surveillance. A spiking experiment was designed in which two viral isolates from distinct RNA viral families (*Togaviridae* and *Reoviridae*) were spiked into clarified subsamples of a pool of 100 mosquitoes (Fig. 1) and sequenced using a library preparation protocol optimised for mosquito samples. The sensitivity and specificity of metatranscriptomic sequencing is assessed and compared with reverse transcription droplet digital PCR (RT-ddPCR) and RT-qPCR. Criteria for positive detection are established, and considerations for laboratory protocol and data analysis are made in an arbovirus surveillance context.

## Materials and Methods

### Mosquito collection

Adult mosquitoes were collected using carbon dioxide-baited encephalitis virus surveillance traps^25^ that were set up overnight and collected the next day. Live mosquitoes were immobilised at −20 °C for 30 minutes and transferred to the laboratory by chilled overnight delivery. Trapping was conducted in November 2016 in Kerang, Victoria, Australia (35.733831 S, 143.925728 E). The mosquitoes were morphologically identified using taxonomic keys^26,27^ on top of a cold plate and stored at −20 °C.

### Virus spike sample preparation

A pool consisting of 100 *Culex (Culex) australicus* Dobrotworsky & Drummond 1953 (part of the *Culex pipiens* complex) mosquitoes was homogenised in 2 mL of Buffer AVL (Qiagen) using 10 glass beads (3 mm diameter; Sigma-Aldrich) and two 1 minute 1,500 rpm cycles on a 2010 Geno/Grinder (SPEX SamplePrep). The homogenised pool was centrifuged for 5 minutes at 15,344 × *g* and six 120 µL subsamples were taken from the supernatant.

Five of the clarified mosquito subsamples (S1–S5) were spiked with differing dilutions of two cell culture-derived viral isolates (Fig. 1). Ross River virus (RRV) strain T48 (family *Togaviridae*, genus *Alphavirus*)^28^ was grown in Vero cells (African green monkey kidney epithelial cells). RRV is a single-stranded, positive-sense RNA virus with a genome approximately 11.8 kb in length^29^. Umatilla virus (UMAV) strain M4941_15 (family *Reoviridae*, genus O*rbivirus*)^30^ was grown in C6/36 cells (*Aedes albopictus* cells). UMAV is a double-stranded RNA virus with a 10-segment genome approximately 19.4 kb in length^31^. The viral load of the RRV isolate was 6.9 × 10^4^ copies/ng of RNA, and for the UMAV isolate it was 1.8 × 10^5^ copies/ng of RNA, as measured by RT-ddPCR (see Supplementary Information for details). The S1 clarified mosquito subsample was spiked with 10 µL of the RRV isolate and the S5 subsample was spiked with 10 µL of the UMAV isolate (1:1 spike dilution). This spike represents the viral load of a pool of 100 mosquitoes containing one mosquito infected with RRV as previously described^32^, which was assembled and measured by RT-qPCR for comparison (Fig. S1). The RRV and UMAV isolates then underwent a serial 20-fold dilution (1:20; 1:400; 1:8,000; 1:160,000) with 1XTE Buffer pH 8 (Sigma-Aldrich). The remaining clarified mosquito subsamples (S2–S5 for RRV; S4–S1 for UMAV) were spiked with 10 µL of inverse concentrations of the serial dilutions (composition of subsamples seen in Table 1), resulting in 140 µL of input material for the nucleic acid extraction.

**Table 1:** Sequencing metadata and assembly information for Ross River virus (RRV) and Umatilla virus (UMAV) spiked mosquito pool samples.

Sample name	S1.1	S1.2	S1.3	S2.1	S2.2	S2.3	S3.1	S3.2	S3.3	S4.1	S4.2	S4.3	S5.1	S5.2	S5.3	Neg
RRV spike dilution	1:1			1:20			1:400			1:8,000			1:160,000			0
UMAV spike dilution	1:160,000			1:8,000			1:400			1:20			1:1			0
Reads (millions)	22.6	23.45	19.1	19.45	17.1	17.05	18.65	20.45	21.05	22.55	18.9	21.2	23.25	19.1	19.45	21.7
Viral reads (%)	16.0	15.4	15.9	15.9	16.9	16.3	15.2	14.6	15.1	14.6	15.6	11.6	17.1	16.9	16.9	15.1
No. of viral contigs	527	536	545	540	551	494	482	557	539	550	511	518	497	491	513	529
RRV contigs	20	16	26	9	25	14	2	6	4	0	3	0	3	0	1	2
UMAV contigs	4	21	6	16	10	6	64	48	52	32	33	33	38	34	48	6
RRV (%)^a^	100.0	100.0	100.0	100.0	97.0	94.5	3.7	11.8	11.1	0.0	5.1	0.0	5.0	0.0	4.4	3.7
UMAV (%)^b^ Total:	5.8	26.0	7.4	21.2	16.2	9.1	92.9	83.0	90.3	98.7	98.8	98.8	98.6	99.1	98.9	7.9
Seg 1 (VP1/RdRp)	0.0	13.8	6.8	7.0	24.4	0.0	82.8	73.3	86.4	100.0	100.0	100.0	100.0	100.0	100.0	0.0
Seg 2 (VP2/T2)	0.0	9.4	0.0	7.3	0.0	8.6	98.0	65.7	93.3	99.0	99.0	98.8	99.0	99.2	99.1	17.4
Seg 3 (VP3)	10.9	29.3	0.0	8.9	9.8	0.0	98.5	93.9	98.6	100.0	100.0	100.0	99.2	100.0	100.0	0.0
Seg 4 (VP4/CaP)	0.0	23.5	0.0	21.7	0.0	0.0	95.3	83.1	94.8	96.1	95.5	96.6	95.4	96.7	96.1	17.0
Seg 5 (NS1/TuP)	29.2	56.6	35.2	49.5	28.2	33.6	100.0	100.0	100.0	100.0	100.0	100.0	100.0	100.0	100.0	0.0
Seg 6 (VP5)	13.8	36.9	13.0	31.1	17.5	37.2	97.9	99.5	99.4	99.4	99.4	99.4	99.6	99.4	99.4	0.0
Seg 7 (NS2/ViP)	0.0	29.5	0.0	19.5	28.5	0.0	93.4	96.0	74.5	99.7	100.0	99.7	99.7	99.7	99.6	35.0
Seg 8 (VP7/T13)	0.0	36.3	0.0	78.5	36.3	0.0	97.8	66.3	87.2	98.4	100.0	100.0	100.0	100.0	100.0	19.1
Seg 9 (VP6/Hel)	0.0	39.4	20.7	23.8	27.3	19.3	95.6	80.9	97.1	96.7	97.5	97.5	97.6	97.5	97.4	0.0
Seg 10 (NS3)	0.0	0.0	0.0	0.0	0.0	0.0	62.7	81.4	45.7	91.6	93.2	90.6	91.4	96.3	93.0	0.0

The reads in millions represent the number of paired, interleaved reads remaining after quality trimming. The viral reads and contigs represent all viruses in the mosquito pool sample. The number of RRV and UMAV contigs is shown, and what percentage of the virus genome is covered by these contigs.

^a^RRV genome length = 11,575 bp. ^b^Total UMAV genome length = 19,318 bp. UMAV segment lengths: Seg 1 = 3,711 bp; Seg 2 = 2,794 bp; Seg 3 = 2,523 bp; Seg 4 = 2,063 bp; Seg 5 = 2,107 bp; Seg 6 = 1,620 bp; Seg 7 = 1,324 bp; Seg 8 = 1,131 bp; Seg 9 = 1,104 bp; Seg 10 = 941 bp.

The sixth 120 µL clarified mosquito subsample had 20 µL of 1XTE Buffer pH 8 added to it and was used as a negative control to ensure the mosquito pool was free of both RRV and UMAV, and to account for any contamination and background noise during sequencing.

### Nucleic acid extraction

Nucleic acid was extracted from the six clarified mosquito subsamples using the QIAamp Viral RNA Mini Kit (Qiagen) according to manufacturers’ instruction, except that carrier RNA was not used. The final elution volume of 80 µL in Buffer AVE was split into three 25 µL aliquots to create technical replicates for each of the spiked clarified mosquito subsamples. This resulted in a total of 15 RNA samples, and one negative control sample (Table 1 and Fig. 1). Due to the double-stranded RNA genome structure of UMAV, all of the RNA was heat-denatured at 100 °C for 1 minute^33^ and immediately placed on ice. The RNA was quantified using a Qubit RNA HS Assay Kit (Thermo Fisher Scientific) and then stored at −80 °C until further analysis.

### Virus spike sample quantification using metatranscriptomic sequencing

Metatranscriptomic sequencing was performed on all 15 spiked mosquito pool samples and the unspiked negative control sample. Sequencing libraries were prepared using the strand-specific NuGEN Ovation Universal RNA-Seq System with custom rRNA depletion, as described by manufacturer’s instructions, unless where noted. The input for library preparation was 2 µL of undiluted heat-denatured RNA (total 165.2–224 ng) as preliminary experiments suggested undiluted RNA yielded more viral reads (Fig. S2A). Library preparation began with transcription of RNA into cDNA with an integrated DNase treatment. The synthesised cDNA was then sheared into 200–400 bp fragments using a S220 focused-ultrasonicator (Covaris). End repair was carried out to generate blunt ends for adaptor ligation and strand selection.

Customised insert dependent adaptor cleavage (InDA-C) ssDNA probes were used to deplete the sample of unwanted mosquito rRNA sequences. A total of 480 InDA-C probes (16–25 bp) were designed by NuGEN based on sequences provided by the authors. Specifically, these included both GenBank rRNA from a variety of mosquito species and highly abundant assembled mosquito contigs from previous metatranscriptomic sequencing of mosquito pools (a FASTA file containing the sequences used for probe design is available on Figshare: 10.6084/m9.figshare.9491258.v1). Preliminary experiments indicated usage of the InDA-C probes at the recommended 500 nM did not effectively deplete mosquito rRNA, however usage at 100 µM resulted in a substantial reduction of mosquito rRNA in both 100 and 1,000 mosquito pool libraries, leading to increases in virus reads (Fig. S2A,B). When used at the 100 µM concentration, the InDA-C probes were shown to reduce mosquito rRNA sequences across a range of species (Fig. S2C). Therefore, the InDA-C probes were used at a 100 µM concentration when preparing the mosquito pool samples.

After customised rRNA depletion the libraries were amplified using 14 PCR cycles and purified. All purification steps were performed using AMPure XP beads (Beckman Coulter). The size of the completed libraries was determined with a 2200 TapeStation using the D5000 ScreenTape assay (Agilent Technologies), and concentration quantified with a Qubit dsDNA HS Assay Kit. The libraries were pooled together in equimolar concentrations, diluted to 10 pM and sequenced on a HiSeq 3000 lane (Illumina) using 2 × 150 bp reads.

### Analysis of metatranscriptomic sequencing data

To detect the spiked viruses in the metatranscriptomic sequencing data, reads from each individual sample were assembled into contigs using Trinity v2.4.0^34^ with the read trimming (–trimmomatic) and normalisation (–normalize_reads) options selected. The assembled contigs were taxonomically classified using BLASTn v2.7.1 + with the NCBI nucleotide (nt) database (acquired 5^th^ February 2019). BLASTn was used to identify the spiked virus contigs as it produced more specific results than BLASTx (Table S1). To determine the breadth of coverage of the spiked viruses, the assembled contigs from the individual sample reads were mapped to one set of full-length RRV and UMAV contigs using BWA-MEM v0.7.17^35^ with default parameters. The BBMap pileup command^36^ was used to calculate what percentage of the virus genome was covered by the contigs.

Cross-validation of the spiked virus detections was performed by mapping trimmed, interleaved reads from the individual samples to the same set of full-length RRV and UMAV contigs with BWA-MEM. Counts were derived from the alignments with the SAMtools v1.9^37^ flagstat command and used to calculate reads per million (RPM). Correlation between RPM and virus spike levels was calculated using a Spearman rank correlation test with R v3.6.1^38^. The read alignments were also used to determine depth of coverage with the SAMtools depth command and visualised with the ggplot2 package v3.1.0^39^ as implemented in RStudio v1.1.463^40^. The BBMap pileup command^36^ was used to calculate average fold coverage of the virus genome by the reads.

The presence of other viruses in the mosquito pool was also assessed by performing a single *de novo* assembly of all the sample reads combined using Trinity. For taxonomical classification, the assembled contigs were compared to the NCBI non-redundant (nr) database (acquired 5^th^ February 2019) using DIAMOND BLASTx v0.9.22.123^41^. BLASTx was used as opposed to BLASTn to enable detection of divergent viruses. Trimmed, interleaved reads from each individual sample were mapped to the assembled contigs from the combined sample reads with BWA-MEM and counts were summed from viral contig alignments to measure the relative abundance of viral families. Contigs were excluded from the count if they were <500 bp long, or if they also matched to the *Culex quinquefasciatus* (part of the *Culex pipiens* complex) genome (GCA_000209185.1) or the cell lines used to grow the RRV and UMAV spiked into the samples (unpublished data).

To investigate the incidence of index cross-talk among the samples, the demultiplexed reads were mapped to the PhiX genome (NC_001422.1) using BWA-MEM. PhiX is an unindexed spike-in control added to Illumina runs prior to sequencing and theoretically should not be present in the demultiplexed sample reads. Furthermore, the raw HiSeq data was re-demultiplexed using bcl2fastq Conversion Software v2.20 (Illumina) with the number of index mismatches changed from 1 to 0. The re-demultiplexed reads were also mapped to the PhiX genome.

The HiSeq FASTQ files used in this study have been uploaded to the NCBI Sequence Read Archive (SRA) under project ID PRJNA559742.

### Re-sequencing of the negative control

To determine the source of contaminating RRV and UMAV reads, the negative control was re-sequenced without the spiked subsamples. First, the negative control library was re-quantified using a Qubit dsDNA HS Assay Kit. The library was then diluted to 10 pM and sequenced on a NovaSeq 6000 System (Illumina) using 2 × 150 bp reads to the same depth as the previously sequenced samples (25 million paired-end reads). The re-sequenced negative control reads were interleaved and mapped to the same set of full-length RRV and UMAV contigs as used in the analysis above.

### Virus spike sample quantification using RT-ddPCR

The viral load of the 15 spiked mosquito pool samples was determined using reverse transcription droplet digital PCR (RT-ddPCR), a highly sensitive method that allows absolute quantification without the need for a standard curve^42^. The primer and probe sequences used can be found in Table S2. Double-quenched probes (Integrated DNA Technologies) were used to reduce RT-ddPCR background and increase signal intensity. The RRV primers and probe sequences were previously published^29^. The UMAV primers and probe were designed using the Primer3 algorithm in Geneious R8^43^ (www.geneious.com) based on an Australian strain of UMAV using the VP2/T2 gene (NC_012755.1) reference sequence^31,33^.

The One-Step RT-ddPCR Advanced Kit for Probes (Bio-Rad) was used to prepare 22 µL reaction mixtures consisting of: 5 µL of Supermix (Bio-Rad); 2 µL of reverse transcriptase (Bio-Rad); 1 µL of 300 mM dithiothreitol (DTT; Bio-Rad); 1.98 µL of each forward and reverse 10 µM virus-specific primer (Sigma-Aldrich); 0.55 µL of 10 µM virus-specific probe (Integrated DNA Technologies); 7.49 µL of UltraPure water (Invitrogen); and 2 µL of heat-denatured RNA. The reaction mixtures were loaded into an AutoDG Instrument (Bio-Rad) to generate droplets using Automated Droplet Generation Oil for Probes (Bio-Rad). The droplets were then used for RT-ddPCR using the following cycling conditions: 50 °C for 60 min; 95 °C for 10 min; 40 cycles of 95 °C for 30 s, 57 °C for 1 min; 98 °C for 10 min. After RT-ddPCR, positive and negative droplets were counted using a QX200 Droplet Reader (Bio-Rad) with FAM and HEX channels. The number of positive and negative droplets were used to calculate the concentration of RRV and UMAV as copies per µL of the final reaction (22 µL in total, including 2 µL of RNA) using QuantaSoft Software (Bio-Rad). Correlation between copies/µL and virus spike levels was calculated using a Spearman rank correlation test with R v3.6.1.

The unspiked negative control sample was also tested for RRV and UMAV using RT-ddPCR with the same specifications as above.

### Virus spike sample quantification using RT-qPCR

In addition to RT-ddPCR, the viral load of the 15 spiked mosquito pool samples was measured using RT-qPCR. A one-step reaction was performed with 25 µL mixtures consisting of: 12.5 µL RT-PCR Buffer (Applied Biosystems); 1 µL of each forward and reverse 10 µM virus-specific primer (Sigma-Aldrich); 1 µL of 3.12 µM virus-specific probe (Integrated DNA Technologies); 1 µL RT-PCR Enzyme Mix (Applied Biosystems); 6 µL of UltraPure water (Invitrogen); and 2.5 µL of heat-denatured RNA. The same primer and probe sequences used for the RT-ddPCR were also used for the RT-qPCR (Table S2). The cycling conditions were as follows: 48 °C for 30 min; 95 °C for 10 min; 40 cycles of 95 °C for 15 s, 57 °C for 45 s. Correlation between Ct values and virus spike levels was calculated using a Pearson correlation test with R v3.6.1.

The unspiked negative control sample was also tested for RRV and UMAV using RT-qPCR, however instead of a probe-based assay, a SYBR-based assay was used to detect potential genetically divergent viral strains. The same reaction volumes and PCR cycle were used as above, however the 1 µL of virus-specific probe was replaced with 1 µL of 10X SYBR Green I (Invitrogen), and a melt curve protocol was added to the end of the cycle: 5 seconds at 0.5 °C increments between 65 °C and 95 °C. The negative control sample melt peak was compared to RRV and UMAV positive control melt peaks to determine if any virus was present.

## Results

### Metatranscriptomic sequencing

A consistent level of sequence reads (mean 20.3 million per library; range 17.1–23.5 million) were obtained across the 15 spiked mosquito pool subsamples and negative control. The percentage of viral reads (mean 15.6%; range 11.6–17.1%) and number of viral contigs (mean 524; range 482–557) were also consistent across all samples (Table 1). Index cross-talk occurred during the sequencing run, with unindexed PhiX reads detectable in every sample (mean 20,944 PhiX reads, range 12,852–30,425; mean 0.05% of sample reads, range 0.03–0.08%). Re-demultiplexing the reads using more stringent parameters did not resolve the index cross-talk (Fig. S3).

**Figure 2:**
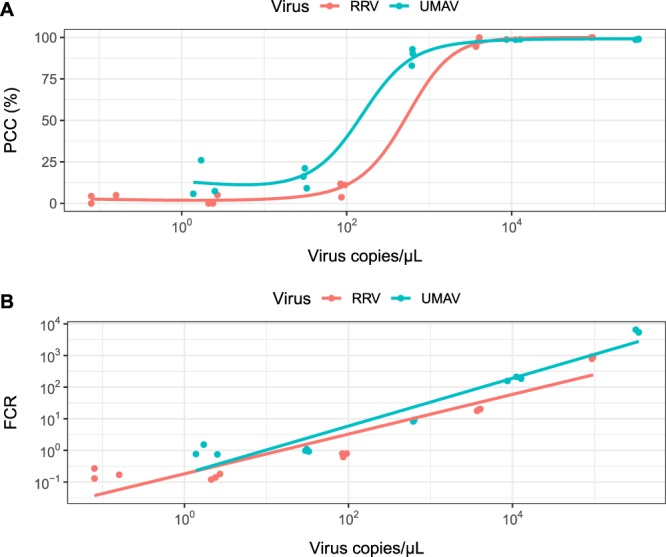
The relationship between copies/µL of Ross River virus (RRV) and Umatilla virus (UMAV) in the spiked mosquito samples and (**A**) percent coverage of the virus genomes by assembled contigs (PCC); (**B**) average fold coverage of the virus genomes by reads (FCR). The virus copies/µL was measured by reverse transcription droplet digital PCR (RT-ddPCR) and represents the final reaction volume (22 µL).

Detection of the spiked viruses using metatranscriptomic sequencing was first evaluated based on the percent genome coverage of the spiked virus by assembled contigs (Percent Coverage by Contigs - PCC) (Table 1). An increase in virus input resulted in an increase in PCC for both RRV and UMAV, reaching a plateau at approximately 2.2 × 10^3^ virus copies/µL (Fig. 2A). The 1:1 spike subsample, which was estimated to represent the RRV load of a pool of 100 mosquitoes containing a single RRV-infected mosquito, had contigs that covered the entire spiked virus genome for both RRV and UMAV. RRV 1:1 spike subsamples had a mean of 20 contigs covering a mean 100% of the genome, whereas UMAV 1:1 spike subsamples had a mean of 40 contigs covering a mean 98.9% of the genome. Contig assembly efficiency differed among the 10 UMAV segments – for example Segment 5 (NS1/TuP) assembled in every spiked sample, but Segment 10 (NS3) only assembled in the three most concentrated UMAV spike subsamples (1:400, 1:20 and 1:1).

**Figure 3:**
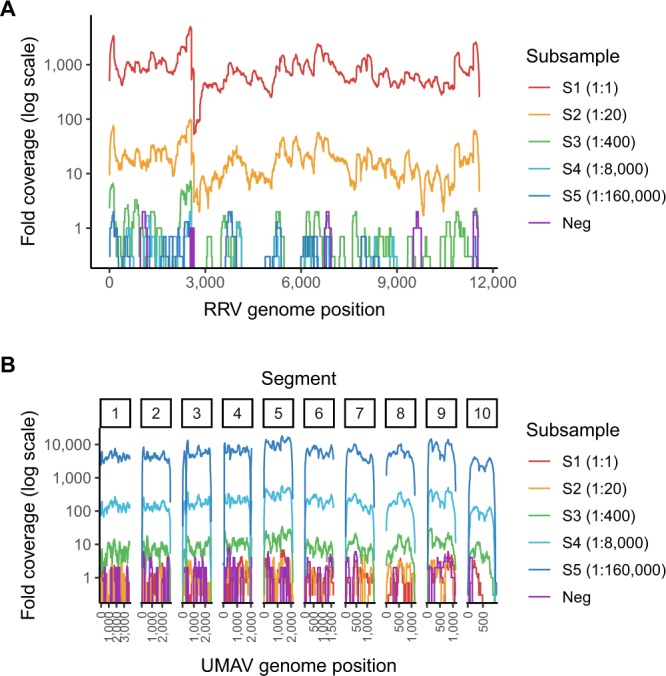
Mean fold coverage of the (**A**) Ross River virus (RRV) genome and (**B**) Umatilla virus (UMAV) genome across the differently spiked subsamples. RRV has a monopartite genome, whereas UMAV has a segmented genome consisting of 10 segments.

Cross-validation of the samples was performed by mapping sample reads to the spiked virus genomes to measure average fold coverage (Fold Coverage by Reads - FCR) (Fig. 3). Like with PCC, an increase in virus input resulted in an increase in FCR for both RRV and UMAV, however FCR does not plateau like PCC does (Fig. 2B). RRV 1:1 spike subsamples had a mean 873.9 fold coverage of the genome, whereas UMAV 1:1 spike subsamples had a mean 5,778.9 fold coverage of the genome.

### Sensitivity of metatranscriptomic sequencing

The PCC from the contig assembly and FCR from the read mapping approach were both used to assess the analytical sensitivity of metatranscriptomic sequencing from virus spiked mosquito pool samples. However, the determination of sensitivity was confounded by the presence of virus contigs (Table 1) and reads (Fig. 3) specific to the spiked viruses in the negative control. The contamination was unique to the metatranscriptomic sequencing as no spiked virus was detected in the negative control by RT-ddPCR or RT-qPCR. Re-sequencing of the negative control library without the spiked subsamples resulted in zero spiked virus reads, suggesting that the contamination occurred during sequencing and not during library preparation.

For the contig assembly, the three lowest RRV spike subsamples (1:400, 1:8,000 and 1:160,000) contained a mean of two contigs covering 4.6% of the RRV genome, and the negative control had 2 RRV contigs covering 3.7% of the genome. Similarly, the two lowest UMAV spike subsamples (1:8,000 and 1:160,000) contained a mean of 10.5 UMAV contigs covering 14.3% of the UMAV genome, and the negative control had six contigs covering 7.9% of the genome.

As for the read mapping approach, the two lowest RRV spike subsamples (1:8,000 and 1:160,000) had a mean 0.17 fold coverage of the genome, while the negative control had 0.11 fold coverage of the genome. For UMAV, the two lowest subsamples (1:8,000 and 1:160,000) had a mean 1.01 fold coverage of the genome, while the negative control had 0.91 fold coverage of the genome.

To address the confounding negative control results, we established a detection criterion based on PCC and FCR (Table 2). To determine if a sample was considered positive for virus spiked into the original mosquito subsample, a normalised PCC ratio (PCC-r) was calculated, where the PCC of the sample (PCC_sample_) is divided by the negative control (PCC_neg_). A sample with a PCC-r ≥ 2 was considered positive, which represents at least twice the level seen in the negative control. The minimum PCC_neg_ is set as 1% to prevent overinflated PCC-r values, which means ≥ 2% PCC_sample_ is required for positive detection of virus. The same calculation and parameters were used for a normalised FCR ratio (FCR-r). In order for a sample to be considered positive for a virus, the PCC-r and FCR-r must both be ≥ 2.

**Table 2:** Criteria established for detection of Ross River virus (RRV) and Umatilla virus (UMAV) in each spiked mosquito subsample.

Subsample	PCC-r		FCR-r	
	RRV	UMAV	RRV	UMAV
S1 (1:1 RRV 1:160,000 UMAV)	26.7 ± 0.0*	1.7 ± 1.4	8,283.3 ± 744.0*	1.1 ± 0.5
S2 (1:20 RRV 1:8,000 UMAV)	25.9 ± 0.7*	2.0 ± 0.8*	181.1 ± 12.1*	1.1 ± 0.1
S3 (1:400 RRV 1:400 UMAV)	2.4 ± 1.2*	11.3 ± 0.7*	7.0 ± 1.0*	9.7 ± 0.6*
S4 (1:8,000 RRV 1:20 UMAV)	0.5 ± 0.8	12.6 ± 0.0*	1.4 ± 0.3	204.2 ± 30.8*
S5 (1:160,000 RRV 1:1 UMAV)	0.8 ± 0.7	12.6 ± 0.0*	1.8 ± 0.7	6,353.4 ± 731.5*

The first criterion is based on the percent genome coverage by contig ratio (PCC-r), which is calculated by dividing the percent coverage of the spiked virus genome by assembled contigs in the sample (PCC_sample_) by the negative control (PCC_neg_). The second criterion is based on the average fold genome coverage by reads ratio (FCR-r), which is calculated by dividing the average fold coverage of the spiked virus genome by reads in the sample (FCR_sample_) by the negative control (FCR_neg_). The threshold value for PCC-r and FCR-r was ≥ 2 (marked by *) and samples need both to be considered as a positive detection of either RRV or UMAV.

The 1:1, 1:20, 1:400, 1:8,000 and 1:160,000 spike subsamples had an RRV PCC-r of 26.7, 25.9, 2.4, 0.5 and 0.8 respectively, and a UMAV PCC-r of 12.6, 12.6, 11.3, 2.0 and 1.7 respectively. The RRV FCR-r for the 1:1, 1:20, 1:400, 1:8,000 and 1:160,000 spike subsamples was 8,283.3, 181.1, 7.0, 1.4 and 1.8 respectively, and the UMAV FCR-r was 1.1, 1.1, 9.7, 204.2 and 6,353.4 respectively. Only the three highest spike subsamples (1:1, 1:20 and 1:400) had both PCC-r and FCR-r ≥ 2 for RRV and UMAV and were therefore considered positive for both viruses.

### Specificity of metatranscriptomic sequencing

Specificity was based on the accuracy of taxonomic classification of the spiked virus contigs assembled for each sample. The BLASTn search of the nt database correctly identified all RRV contigs from the individual samples as RRV, whereas all but two (0.4%) of the UMAV contigs were correctly identified as UMAV (Table S1B). Those two contigs were identified as Koyama Hill virus (KHV), which is also a member of the *Umatilla virus* species^44^. On closer inspection of the two contigs it was found the sequences matching KHV were in an untranslated region (UTR), and the UMAV reference in the nt database did not contain UTR sequences.

### Comparison of virus detection methods

To compare the three virus detection methods: RPM from the metatranscriptomic sequencing results; the copies/µL measurement from the RT-ddPCR; and the cycle threshold (Ct) value from the RT-qPCR were used (Table 3). Virus spike levels positively correlated with RPM (*R* = 0.927, *p* = < 0.001) and copies/µL (*R* = 0.982, *p* = < 0.001), and negatively correlated with Ct (*R* = −0.76, *p* = 0.002). The lowest concentration spike subsample (1:160,000) was detectable by both RT-ddPCR and RT-qPCR, with mean 0.1 copies/µL and Ct 38.8 for RRV, and mean 1.9 copies/µL and Ct 34.0 for UMAV. Based on the PCC-r and FCR-r criterion, only the three highest spike subsamples were considered positive for RRV and UMAV (1:1, 1:20 and 1:400). The lowest of these (1:400) corresponded to mean 1.6 RPM, 88.6 copies/µL and Ct 27.8 for RRV, and mean 30.6 RPM, 625.1 copies/µL and Ct 26.2 for UMAV.

**Table 3:** Comparison of Ross River virus (RRV) and Umatilla virus (UMAV) quantification in the spiked mosquito subsamples and negative control using metatranscriptomic sequencing, reverse transcription droplet digital PCR (RT-ddPCR) and quantitative PCR (RT-qPCR).

	Sequencing (RPM)		RT-ddPCR (copies/µL)		RT-qPCR (Ct)	
Subsample	RRV	UMAV	RRV	UMAV	RRV	UMAV
S1 (1:1 RRV 1:160,000 UMAV)	1,785.7 ± 65.4*	3.5 ± 1.2	93,766.7 ± 1,517.3	1.9 ± 0.5	17.9 ± 0.1	34.0 ± 1.1
S2 (1:20 RRV 1:8,000 UMAV)	46.8 ± 1.3*	4.0 ± 0.1	3,851.2 ± 147.8	31.3 ± 1.2	23.3 ± 0.1	30.6 ± 0.1
S3 (1:400 RRV 1:400 UMAV)	1.6 ± 0.1*	30.6 ± 2.0*	88.6 ± 4.8	625.1 ± 7.2	27.8 ± 2.0	26.2 ± 0.05
S4 (1:8,000 RRV 1:20 UMAV)	0.3 ± 0.1	619.7 ± 91.9*	2.4 ± 0.2	10,860.0 ± 1,664.8	34.1 ± 0.1	21.8 ± 0.1
S5 (1:160,000 RRV 1:1 UMAV)	0.4 ± 0.2	19,518.8 ± 281.4*	0.1 ± 0.04	336,466.7 ± 12,922.9	38.8 ± 0.9	16.6 ± 0.1
Negative control	0.1	1.4	0	0	0	0

The sequencing results are shown as mapped reads per million (RPM), with the subsamples considered positive marked by an asterisk (based on having percent coverage by contig ratio (PCC-r) and average fold coverage by reads ratio (FCR-r) both ≥ 2). The RT-ddPCR measurement refers to copies per µL of the final reaction (22 µL in total). Aside from the negative control, all results are shown as mean with one standard deviation based on three technical replicates.

### Detection of other viruses

**Figure 4:**
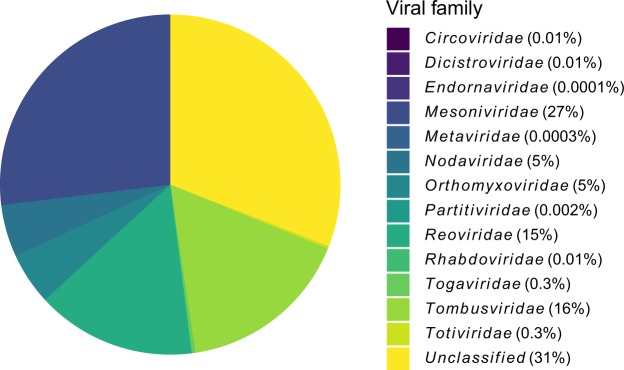
The viral family composition of the pool of 100 *Culex australicus* mosquitoes, shown as percentage of total read counts for each family.

In addition to the two spiked viruses, metatranscriptomic sequencing revealed the presence of other viruses in the pool of 100 *Cx. australicus* mosquitoes (Fig. 4). The most abundant assembled virus contigs were classified as *Mesoniviridae* (27%), *Tombusviridae* (16%) and *Reoviridae* (15%) or were unclassified (31%). Nineteen previously characterised viruses were present in the pool (Table S3) all of which have been detected in mosquito samples and are currently considered to be insect-specific.

## Discussion

In this study, we used a spiking experiment to investigate the analytical sensitivity and specificity of a metatranscriptomic pipeline in detecting two RNA viruses with differing genome structure in a pool of 100 *Cx. australicus* mosquitoes (Fig. 1). The metatranscriptomic pipeline successfully sequenced the full-length genome of both viruses in the spiked subsample that corresponded to a biologically relevant viral load representing a single RRV-infected mosquito in a pool of 100 mosquitoes (1:1 spike dilution). Detection of RRV in a pool of 1,000 mosquitoes containing one RRV-infected mosquito was also demonstrated (Fig. S2B). This level of sensitivity was achieved by using a customised mosquito rRNA depletion, which helped to achieve a higher portion of viral sequencing reads (11.7–17.3%) compared to other metatranscriptomic studies where mosquito pool samples had as little as <1% viral reads^45–47^. Notably, the rRNA depletion was effective only when a higher concentration of the customised probe mixture was used than advised in the manufacturer’s instructions (Fig. S2A,B). It is possible this is due to the particularly high level of host RNA when using a large pool of mosquitoes as starting material. Other approaches that helped to increase sensitivity were the use of undiluted RNA as input for library preparation (Fig. S2A) and RNA heat-denaturation to improve detection of dsRNA genomes (data not shown). All these approaches are recommended to improve sensitivity when performing metatranscriptomic sequencing of mosquito traps for arbovirus surveillance.

In order to increase accuracy, detection of the spiked virus genomes in the metatranscriptomic data was first performed using a contig assembly approach from which Percent Coverage by Contigs (PCC) was derived, and then cross-validated with read mapping to the virus genomes, from which Fold Coverage by Reads (FCR) was derived. While there was a strong relationship between PCC/FCR and the viral copies/µL (Fig. 2A,B), the presence of contigs and reads specific to the spiked viruses in the negative control confounded detection at lower viral loads (Table 1 and Fig. 3). Re-sequencing of the negative control library returned zero spiked virus reads, indicating that the contamination occurred during sequencing and not during library preparation. It is possible the contaminating reads are a result of index cross-talk, which occurs when reads are misassigned due to incorrect matching of the indexes used to multiplex samples^48^. The presence of PhiX in the sample reads also indicate index cross-talk occurred, since the PhiX spike-in is unindexed and therefore should not be present in any of the demultiplexed samples. Index cross-talk can be caused by spreading of signal on flow cells, sequencing errors introduced during bridge amplification, improper cluster resolution and misread indexes^48^. The rate of index cross-talk increases with the use of Illumina patterned flow cells, and also single indexes^49^, both of which were employed in this study. Using unique dual indexes to multiplex samples has been shown to significantly reduce index cross-talk, thereby increasing the sensitivity of sequencing^48,49^. Therefore, the use of both negative controls and unique dual indexes is recommended when using metatranscriptomics for sensitive applications such as surveillance in order to improve detection and dependability of the results.

To account for the contaminating reads in the negative control, we established a detection criterion where the PCC_sample_ and FCR_sample_ for a virus is divided by the PCC_neg_ and FCR_neg_, respectively, to produce normalised ratios (PCC-r and FCR-r). Both ratios must be ≥ 2 for that sample to be considered positive for a virus. Using this criterion, only the three highest spike subsamples (1:1, 1:20 and 1:400) were positive for both RRV and UMAV (Table 2). The maximum FCR-r value for RRV was higher than for UMAV (8,283.3 vs. 6,353.4), which was due to the negative control containing less RRV reads than UMAV reads (FCR_neg_ 0.11 vs. 0.91). This pattern was also present in the RRV and UMAV PCC-r and PCC_neg_ values. The higher UMAV contamination in the negative control was due to the subsamples having higher concentrations of UMAV than RRV (Table 3), leading to increased index cross-talk^50^. The higher UMAV concentrations also meant that the PCC plateaued earlier for UMAV than for RRV (Fig. 2A). The PCC-r and FCR-r values give an indication of the virus genome assembly and coverage, and virus concentration present in a sample, respectively, while accounting for contamination in the negative control. Patterns in these values can be useful for surveillance, for instance, a high PCC and low FCR suggests a virus is present but at low concentration. Conversely, a low PCC and high FCR could be indicative of a related virus or erroneous reference genome^51^. This approach is dependent on complete genome sequences in the reference database for accuracy, and care needs to be taken when analysing segmented viruses to ensure coverage is calculated for the whole genome and not just one segment. As with any detection tool, it is recommended that any viruses of public health concern detected by metatranscriptomic sequencing are confirmed using alternative virus detection methods such as PCR^52^.

Further studies utilising simulated samples with a finer scale of virus concentration and more negative samples will allow validation of the cut-off values for the PCC-r and FCR-r criterion using a robust statistical-based approach. The proposed value of ≥ 2 means positive detections are at least twice the level seen in the negative control, which has been previously used in other diagnostic tests such as PCR^53^ and ELISA^54^, however remains arbitrary when based on only one negative control sample. Future metatranscriptomic sequencing of mosquito pools that are known to be positive for arboviruses can also be used for further evaluation of the criterion and will improve this approach as a routine surveillance tool.

When investigating the assembly of the 10 UMAV genome segments, we found that certain segments assembled in every sample, while other segments, regardless of segment size, only assembled in higher concentration spike samples (Table 1). When reads were mapped to the UMAV genome all 10 segments had similar coverage for each spiked subsample (Fig. 3B), suggesting the difference in assembly efficiency does not have to do with the availability of the segments in the samples but rather with the contig assembly analysis. Often the inability to detect all of a segmented virus genome suggests the segments are highly divergent from previously sequenced viruses^55^, however the reference genome for the UMAV strain spiked into the mosquito subsamples was in the NCBI nt database used for analysis. Segment 5 (NS1/TuP) was the most frequently assembled segment (29.2–100% PCC across all samples) and interestingly, has the longest UTR sequence that has been recorded for an orbivirus^31^. It is possible the varying lengths of the UTR sequences for each segment may have affected the assembly^56^. Further work to investigate this anomaly could include the comparison of different transcriptome assemblers when working with segmented genomes.

A consistently high specificity was achieved by assembling and taxonomically identifying the spiked viruses, with 100% of RRV and 99.6% of UMAV contigs identified correctly. The misidentification of two UMAV contigs as KHV (also a member of the *Umatilla virus* species) occurred because the UMAV genome in the nt database did not contain any UTR sequences, demonstrating how incomplete reference databases can lead to errors. The specificity was also dependent on the taxonomic classification approach – when BLASTx (translated nucleotide query to protein database) was used instead of BLASTn (nucleotide query to nucleotide database), it led to a decrease in specificity (Table S1B). BLASTx is commonly used in metatranscriptomic data analysis as it can detect divergent sequences which enables novel virus discovery, however BLASTn produces less erroneous results and therefore may be more suited for known pathogen identification^57^. Often studies take a combined approach that utilises both nucleotide and protein information to achieve more accurate and sensitive virus classification^58–60^.

Metatranscriptomic sequencing was not as sensitive as RT-ddPCR and RT-qPCR in detecting the spiked viruses, with both PCR methods successfully detecting RRV and UMAV in all of the spiked mosquito pool subsamples without producing background noise in the negative control (Table 3). Eliminating the contaminant sequences in the negative control would improve the sensitivity of metatranscriptomic sequencing. However, other factors can also affect sensitivity, including the type of sample being used. Metatranscriptomic sequencing has reached a virus detection limit similar to diagnostic qPCR when liquid biological samples are being used, such as blood^61,62^, nasopharyngeal swabs^63,64^ or clarified cell culture supernatant^65^. However, when complex samples such as sewage^1,66^ or plant tissue^67^ are used, metatranscriptomic sequencing is considerably less sensitive. Despite this reduced sensitivity, it is important to note metatranscriptomic sequencing can detect multiple regions, if not the entire virus genome (Fig. 3), whereas PCR targets only a small region. Acquiring more genomic information enables detection of viruses that may evade PCR due to sequence divergence in the diagnostic region and can also be used for molecular epidemiology to gain insight into viral emergence and spread during an outbreak. The utility of this approach was recently evidenced in Nigeria during a Lassa fever outbreak, where metatranscriptomic sequencing on a MinION sequencer enabled simultaneous detection and characterisation of Lassa virus, a highly variable RNA virus that poses difficulties for PCR-based diagnostics^4^. The use of whole genome information is highly beneficial for surveillance not only to describe the diversity of viruses circulating, but also to understand where they came from, how they will be transmitted, and how different strains have evolved over time.

The nontargeted nature of metatranscriptomics meant that not only were the whole genomes of the spiked viruses sequenced, so were other viruses present in the pool of 100 *Cx. australicus* mosquitoes. *De novo* assembly revealed a variety of viral families (Fig. 4), which included 19 previously characterised viruses (Table S3). These results are consistent with prior metatranscriptomic studies, with 15 of the viruses identified in Australian mosquitoes, and 11 of those from the Shi *et al*. study^11^. This is the first time Culex circovirus-like virus, Culex Hubei-like virus, Culex-associated Tombus-like virus and Yongsan picorna-like virus 2 have been detected in Australia. The detection of a circovirus (ssDNA virus) confirms that the metatranscriptomic protocol used is capable of sequencing DNA viruses, despite being targeted at RNA viruses. With DNase-treated RNA as the input material it is possible this is mRNA produced by the circovirus, and it could also be DNA if the DNase treatment was not 100% efficient^68^. A recent study on contaminating viral sequences in virome data suggests circovirus-like viruses are a common contaminant derived from laboratory components^69^. Other types of DNA viruses would need to be tested to determine if this protocol can detect both RNA and DNA without separate nucleic acid library preparations. Whilst the known viruses identified in this pool of mosquitoes are not known to cause disease in mammalian cells, the ability to detect these viruses without targeting them highlights the value of metatranscriptomic sequencing in arbovirus surveillance.

The wealth of information provided by metatranscriptomic sequencing enhances arbovirus surveillance, however this tool needs to be affordable in order to be broadly utilised in surveillance programs. Processing a sample with the same commercial kits and depth of sequencing used in this study costs approximately AUD$230. Over half of this cost is attributed to the library preparation with customised rRNA depletion, and could be reduced by using a cheaper kit (e.g. NEBNext Ultra II RNA) and an in-house depletion method, such as the Cas9-based approach described in Gu *et al*.^70^. The second largest cost is sequencing, with the ~20 million reads per sample used in this study costing approximately AUD$100 using an Illumina NovaSeq sequencer^71^. This depth of sequencing enabled detection of RRV in the 1:400 spike subsample, which is equivalent to 1 positive mosquito in 40,000, therefore the sequencing depth and cost could be halved whilst remaining considerably sensitive. These suggested changes lower the overall cost per sample to approximately AUD$110. This cost does not include labour time, which amounts to approximately three days for the nucleic acid extraction and library preparation of 32 samples. Automation of some of the steps could increase the number of samples processed simultaneously. The NovaSeq run time is 40 hours^71^, resulting in a week turnaround time. Due to the cost and time involved, metatranscriptomic sequencing is currently suited as an additional tool to routine surveillance, providing in-depth information on viral activity in mosquito populations at regular intervals throughout the season, perhaps on a monthly basis. It is likely the time and cost associated with metatranscriptomic sequencing will decrease in the future, allowing it to be used more routinely.

This study has provided information on the sensitivity and specificity of metatranscriptomic sequencing for detection of arboviruses in large pools of mosquitoes, which is essential for the incorporation of this technique into arbovirus surveillance programs. Metatranscriptomic sequencing successfully detected a virus in a pool of 100 mosquitoes at biologically relevant levels, and also in a pool of 1,000 mosquitoes (Fig. S2B). While metatranscriptomic sequencing was less sensitive than diagnostic gold standard approaches such as RT-qPCR and RT-ddPCR, it provided more in-depth information by spanning the entire virus genome, and detecting all viruses present in the mosquito pool. Choices made during the laboratory process and bioinformatic analysis affected the sensitivity and specificity of virus detection, and therefore standardised protocols for both processes need to be established for routine use of metatranscriptomic sequencing. The criterion for positive detection of a virus established in this paper is one example of a process that can be applied to produce comparable results, which also accounts for potential contamination found in the negative control. Further work utilising wild caught mosquitoes from diverse populations will help to establish metatranscriptomic sequencing as a tool that can broaden the capabilities of arbovirus surveillance.

## Supplementary information

Supplementary Information

## Data Availability

The sequences used for the customised mosquito rRNA probe design are available as a FASTA file on Figshare: 10.6084/m9.figshare.9491258.v1. The unprocessed FASTQ files from the Illumina HiSeq are available on the NCBI SRA Database under project ID PRJNA559742.
