# Supplementary material for: Sensitivity and specificity of metatranscriptomics as an arbovirus surveillance tool
Source: Sci Rep. 2019 Dec 18;9:19398. doi: 10.1038/s41598-019-55741-3 (PMC6920425; doi:10.1038/s41598-019-55741-3)
Supplement: Supplementary file 1 — Supplementary Information [file 41598_2019_55741_MOESM1_ESM.docx]

# Supplementary Information

# Sensitivity and specificity of metatranscriptomics as an arbovirus surveillance tool

Jana Batovska,^a,b^* Peter T. Mee,^a^ Stacey E. Lynch,^a^* Tim I. Sawbridge,^a,b^ and Brendan C. Rodoni,^a,b^

^a^ Agriculture Victoria Research, AgriBio Centre for AgriBioscience, Bundoora, Victoria, Australia

^b^ School of Applied Systems Biology, La Trobe University, Bundoora, Victoria, Australia

* Address correspondence to Jana Batovska, jana.batovska@agriculture.vic.gov.au or Stacey E. Lynch, stacey.lynch@agriculture.vic.gov.au.

**Estimating virus isolate concentration using RT-ddPCR**

The virus isolates that were spiked into the mosquito homogenate subsamples were first quantified using reverse transcription droplet digital PCR (RT-ddPCR). The method and calculations used are described below using the Ross River virus (RRV) isolate as an example.

***Lab method***

RNA was extracted from the RRV isolate using a QIAamp Viral RNA Mini Kit and quantified using an RNA HS assay on the Qubit:

- Qubit reading = 4.58 ng/µL

The RNA was then diluted 1:1,000 for input into RT-ddPCR, which was performed using a One-Step RT-ddPCR Advanced Kit for Probes. The diluted RNA input was 2 µL and the sample mix was 22 µL in total.

- RT-ddPCR reading = 69.035 copies/µL

In order to normalise it with the Umatilla virus (UMAV) isolate, the RRV isolate was diluted by a factor of 2.4 and spiked into the S1 mosquito homogenate subsample. The calculation used to estimate the concentration of the spiked RRV isolate is shown below.

***Calculation***

Start with RT-ddPCR reading:

- 69.035 copies/µL

Number of copies of cDNA/RNA in the entire 22 µL reaction:

- 69.035 x 22 = 1,518.774 copies
- Note: The assumption here is that conversion efficiency from RNA to cDNA is 100%.

Concentration of input RNA (2 µL used):

- 1,518.774 / 2 = 759.387 copies/µL

Concentration of RNA before 1:1,000 dilution:

- 759.387 x 1,000 = 759,387 copies/µL

Calculate copies/ng of RNA:

- Qubit reading = 4.58 ng/µL
- 759,387 / 4.58 = 165,805 copies/ng of RNA
- Note: The assumption here is that all the RNA measured by the Qubit belongs to RRV.

Adjust for final dilution:

- 165,805 / 2.4 = 68,952.88 copies/ng of RNA

= 6.9 x 10^4^ copies/ng of RNA

**Table S1:** A comparison of the number of assembled contigs from all individual samples classified as **A)** ‘*Togaviridae*’ or **B)** ‘*Orbivirus*’ using a BLASTn search of the NCBI nucleotide database, and a BLASTx search of the non-redundant database. Closely related viruses are grouped together by different colours.

**A)**

| **BLASTn results** | | **BLASTx results** | |
| --- | --- | --- | --- |
| **No. of contigs** | **Taxonomic ID** | **No. of contigs** | **Taxonomic ID** |
| 131 | Ross River virus | 123 | Ross River virus |
|  |  | 1 | Getah virus |

**B)**

| **BLASTn results** | | **BLASTx results** | |
| --- | --- | --- | --- |
| **No. of contigs** | **Taxonomic ID** | **No. of contigs** | **Taxonomic ID** |
| 339 | Anopheles hinesorum orbivirus | 369 | Anopheles hinesorum orbivirus |
| 246 | Anopheles annulipes orbivirus | 236 | Anopheles annulipes orbivirus |
| 449 | Umatilla virus M4941_15 | 54 | Morris orbivirus |
| 2 | Koyama Hill virus | 339 | Umatilla virus M4941_15 |
|  |  | 51 | Stretch Lagoon orbivirus |
|  |  | 48 | Koyama Hill virus |
|  |  | 4 | Umatilla virus USA1969/01 |
|  |  | 1 | Minnal virus |

**Table S2**: Primer and probe sequences used for reverse transcription qPCR and ddPCR quantification of Ross River virus (RRV) and Umatilla virus (UMAV) in clarified mosquito subsamples.

| **Name** | **Sequence (5’–3’)** | **Amplicon size (bp)** | **Gene** | **Reference** |
| --- | --- | --- | --- | --- |
| RRVE2F | ACGGAAGAAGGGATTGAGTACCA | 67 | E2 | (Hall et al., 2011) |
| RRVE2R | TCGTCAGTTGCGCCCATA |  | E2 | (Hall et al., 2011) |
| RRV_E2_ZEN_IB | 56-FAM/CAACAACCC/ZEN/GCCGGTCCGC/3IABkFQ | N/A | E2 | (Hall et al., 2011) |
| UMAV_84_F | CAGAGAGATGACTATCGACG | 84 | Seg 2 (VP2/T2) | This study |
| UMAV_84_R | TTGTAGGTTCCGATCATAGG |  | Seg 2 (VP2/T2) | This study |
| UMAV_84_ZEN_IB | 56-FAM/CACAAGCAT/ZEN/GGTTACGTACATATTC/3IABkFQ | N/A | Seg 2 (VP2/T2) | This study |

**Table S3:** Known viruses present in the pool of 100 *Culex australicus* mosquitoes as identified by de novo assembly. The length of the assembled contigs is shown along with the amino acid percentage identity (AA PI%) to the reference sequence.

| **Viral family** | **Virus** | **Contig length/s (bp)** | **AA PI%** |
| --- | --- | --- | --- |
| *Circoviridae* | Culex circovirus-like virus† | 1,256-1,472 | 96.9-97.2 |
| *Mesoniviridae* | Ngewotan virus* | 20,244 | 99.4 |
| *Nodaviridae* | Culex Hubei-like virus† | 668 | 97.6 |
| *Orthomyxoviridae* | Wuhan Mosquito Virus 6* | 921-1.554 | 99.7-100 |
| *Reoviridae* | Anopheles annulipes orbivirus | 4,414 | 99.6 |
| *Rhabdoviridae* | Beaumont virus | 4,395 | 96.8 |
| *Tombusviridae* | Culex-associated Tombus-like virus† | 1,752 | 99.6 |
| *Totiviridae* | Australian Anopheles totivirus | 923-6,142 | 67.9-97.3 |
| Unclassified | Castlerea virus | 9,456 | 100 |
| Unclassified | Culex mononega-like virus 1* | 1,681-5,974 | 99.8-100 |
| Unclassified | Culex mononega-like virus 2* | 8,668-13,297 | 99.8-100 |
| Unclassified | Culex negev-like virus 1* | 11,002 | 99.6 |
| Unclassified | Culex negev-like virus 3* | 1,494-7,299 | 99.6-100 |
| Unclassified | Culex phasma-like virus* | 1,886-5,205 | 100 |
| Unclassified | Culex rhabdo-like virus* | 11,455 | 100 |
| Unclassified | Hubei chryso-like virus 1* | 3,149-3,370 | 99.8-100 |
| Unclassified | Hubei reo-like virus 7* | 3,717 | 96.3 |
| Unclassified | Yongsan picorna-like virus 2† | 9,781 | 96.4 |
| Unclassified | Zhejiang mosquito virus 3* | 2,834 | 96.7 |

***** Viruses previously detected in Western Australia mosquitoes (Shi et al., 2017).

† Viruses detected for the first time in Australia.

**Fig. S1:** The reverse transcription quantitative PCR (RT-qPCR) amplification curves for Ross River virus (RRV) in: **A)** A pool of 100 *Culex australicus* mosquitoes containing a single RRV-infected mosquito (run in triplicate); **B)** The RRV-spiked mosquito subsample (S1 – S5) replicates (run in duplicate). The S1 subsample (1:1 spike dilution) is representative of the viral load found in A) and is marked on the amplification plot.


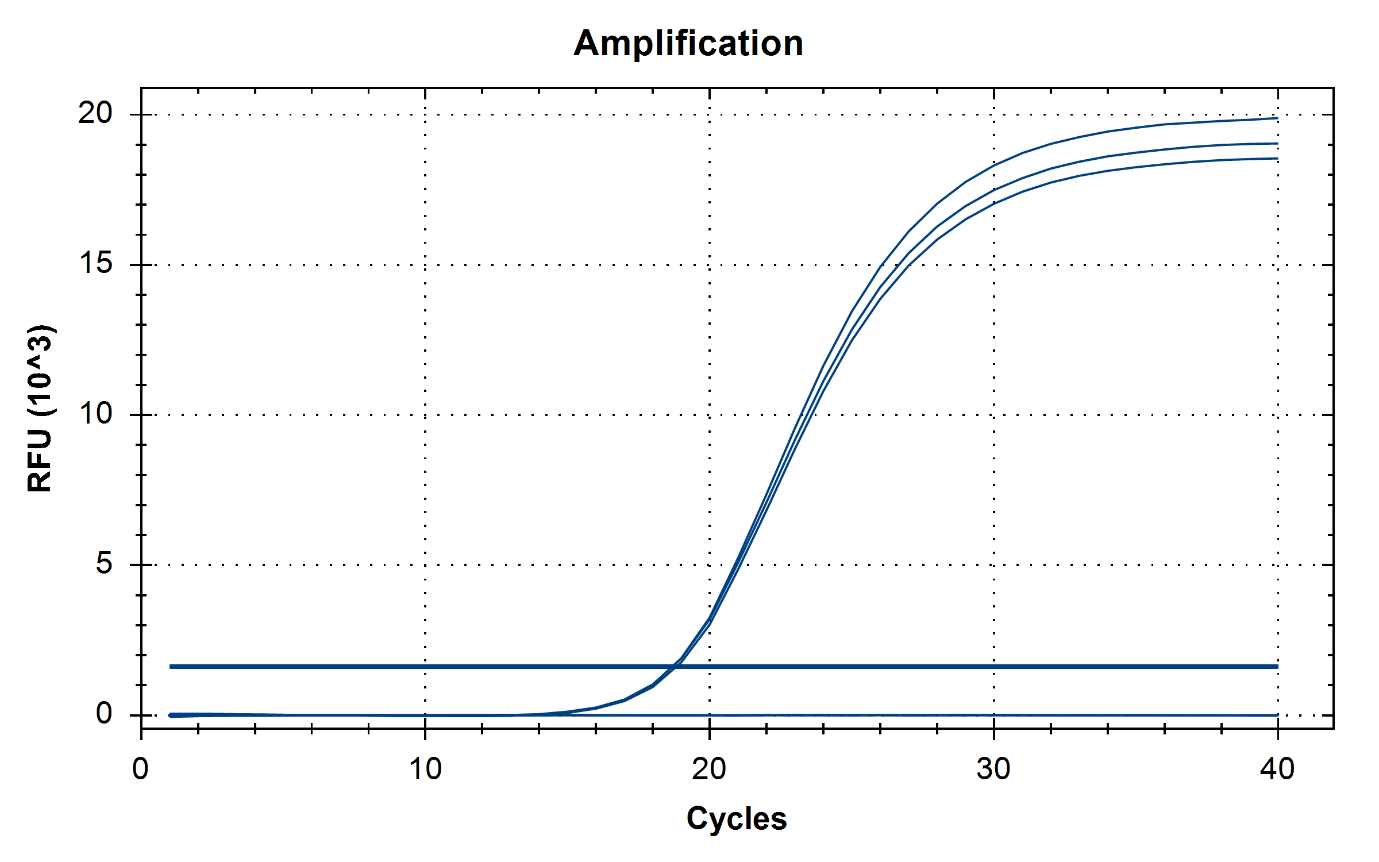
**A)**

**B)**


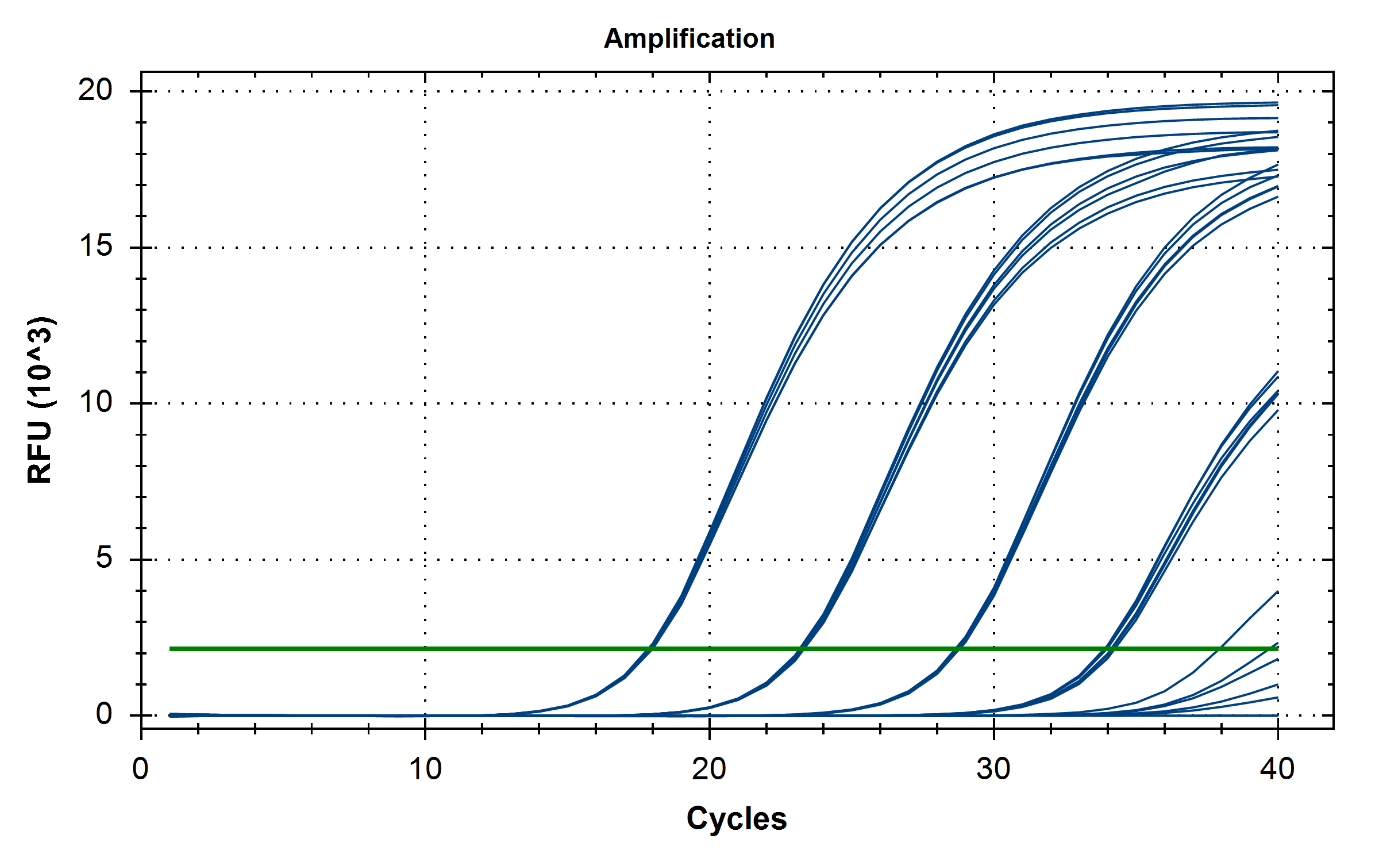


**S1**

**Fig. S2**: Percentage of normalised reads mapping to mosquito ribosomal RNA (rRNA) from:
**A)** A pool of 100 mosquitoes (99 *Culex australicus* and one *Aedes notoscriptus* infected with Ross River virus). NuGEN Ovation Universal RNA-Seq System library preparation was performed with combinations of undiluted (82.6 – 112 ng/µL) and diluted (30 ng/µL) input RNA, and customised rRNA depletion probe mixture added at the recommended 500 nM (blue) and 100 µM (red). The number of Ross River virus reads in each sample is displayed in white text. Reads for each sample were normalised to 600,000.


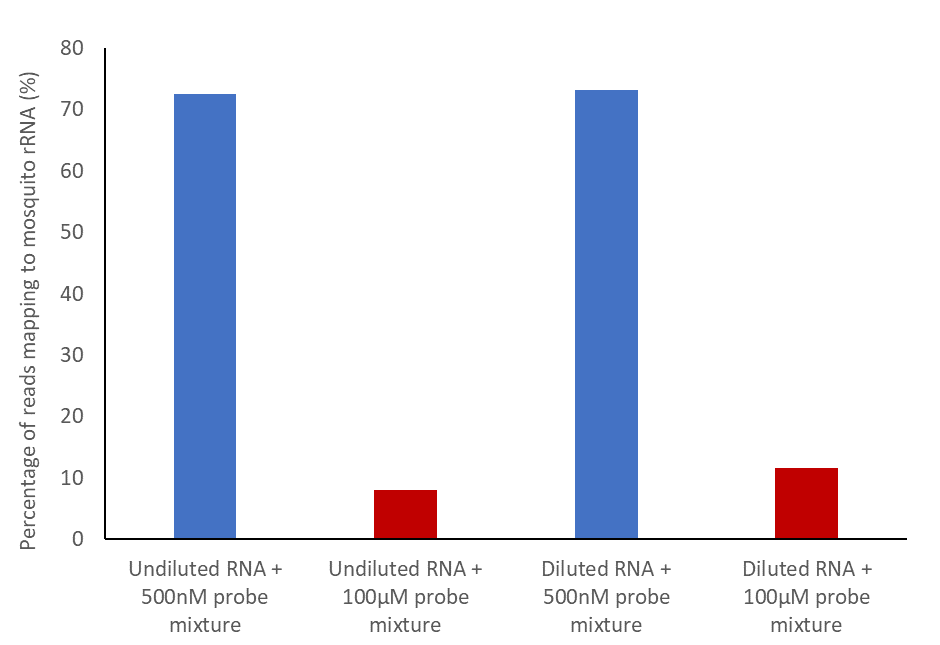


**123**

**762**

**293**

**30**

**B)** A pool of 1,000 mosquitoes (999 *Cx. australicus* and one *Ae. notoscriptus* infected with Ross River virus). NuGEN library preparation was performed without the customised rRNA depletion probe mixture (red), and with the customised rRNA depletion probe mixture added at the recommended concentration of 500 nM (blue) and at 100 µM (green). The number of Ross River virus reads in each sample is displayed in white text. Reads for each sample were normalised to 600,000.


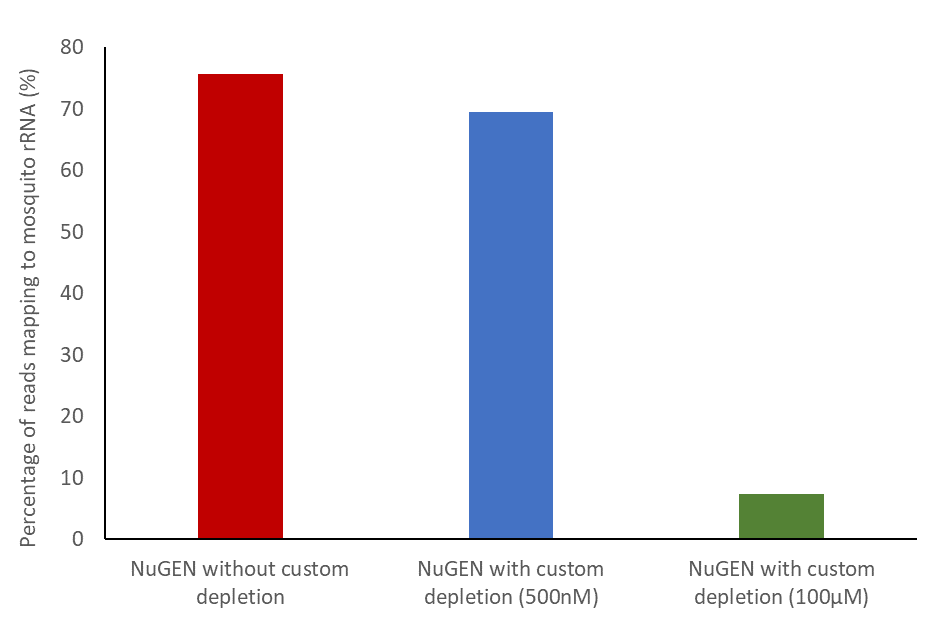


**0**

**32**

**124**

**C)** Individual *Cx. quinquefasciatus*, *Cx. australicus*, and *Ae. notoscriptus* mosquitoes. Each sample was prepared with (red) and without (blue) customised rRNA depletion. The depletion was performed with customised probe mixture added at 100 µM. Reads for each sample were normalised to 800,000.

**Fig. S3**: Number of reads mapping to the PhiX genome from each set of FASTQ files output by the HiSeq sequencer. The initial read demultiplexing allowed one index mismatch, whereas the re-demultiplexed reads had no index mismatches.
